# Study on Tourism Consumer Behavior Characteristics Based on Big Data Analysis

**DOI:** 10.3389/fpsyg.2022.876993

**Published:** 2022-05-02

**Authors:** Muyi Gan, Yao Ouyang

**Affiliations:** Shunde Polytechnic, Foshan, China

**Keywords:** big data, tourism consumer behavior, secondary consumption, scenic spot analysis model, Daming Mountain of Nanning

## Abstract

In terms of scenic marketing, big data research also plays an important role in the precise marketing of scenic spots. This paper has focused on the big data related to scenic spots as the research object, explores the relationship between various subdivision big data and the number of tourists in scenic spots, and investigates the difference and influence of the consumption behavior of the secondary consumption items in the scenic area, to find the potential of the scenic area’s business growth and to promote the continuous and stable growth of the scenic area’s sales and tourism economy. Using the relevant theories and analysis methods, such as consumer behavior, big data, and tourism consumer behavior, the content mainly focuses on the establishment of the analysis model of the number of tourists in the scenic spot, the data collection, the estimation of the model parameters, the various types of big data, the calculation of the contribution rate of the data to the number of tourists in the scenic spot, and the difference analysis of the secondary consumption items of different types of tourists in the scenic spot. Results show that a multi-objective analysis model is established based on the relevant econometric theories, and an optimization plan is proposed after the multicollinearity diagnosis of the model; to establish a data envelopment analysis (DEA) model of the difference and influence of different types of tourists’ consumption behavior in scenic spots and study the consumption behavior characteristics of different types of tourists when they purchase secondary consumption items in scenic spots; the econometric model is used to analyze the big data, adjust the linear relationship of some variables, then adopt the method of gradually adding variables combined with the consumer theory, and finally determine the number of daily tourists as the explained variable, the number of internet protocol (IP), Baidu index, and the virtual value of the weekend, dummy variables for variables, bounce rate, and air pollution as explanatory variables.

## Introduction

Under the background of the new era of mass tourism and global warming, tourism has ushered in a new period of development opportunities, gradually developed into a rigid demand of consumers, and has become a key area that the government, tourism enterprises, scientific research, and media have paid close attention to innovating (Hassan, 2022). Statistics from the data center of the Ministry of Culture and Tourism show that in the third quarter of 2017, the domestic tourism market still maintained a rapid growth rate, with 1.270 billion tourists, a year-on-year increase of 12.59%; tourism revenue was 1,266.541 billion yuan, a year-on-year increase of 17.81%. In the context of the digital age, the networking of tourism activities has made tourists the creators of Internet content (Saarinen et al., 2021). Through online travel notes or evaluations, tourists can share their itineraries and travel experiences, evaluate tourist destinations or services anytime, and provide a reference for potential tourists. Scholars have gradually adopted user-generated content (UGC) data to conduct tourism research (Chen et al., 2021). Compared with traditional data, such as questionnaires and interviews source, UGC data reduce the subjective and design limitations of traditional survey methods, such as questionnaires and interviews, making the research results more objective and practical, low cost, and easy availability. Therefore, UGC data, such as online travel notes and online travel reviews, have become the main research tools for more and more domestic and foreign scholars and have also become an important data source for tourism consumer behavior research (Gajdošík, 2021). Consumer behavior research has always been a hot issue in tourism and marketing circles. Tourism plays a pivotal role in the tourism market (Shih, 2006). However, in the field of academic research, the research process on summer tourism at home and abroad is relatively lacking. There is no direct and clear concept of summer tourism in foreign research, and most of their research focuses on tourism climate and the impact of climate change on tourism decision-making (Caber and Albayrak, 2016).

The proposal of big data was originated from an article published in the special issue of the journal *Nature* in September 2008, which formally proposed the concept of big data (Inmon, 2008). In March 2012, to improve the government’s ability to mine key data and trends from massive coarse-grained data, the U.S. government announced the “Big Data R&D Plan” to enhance the federal government’s ability to collect massive data, analyze and extract information, and meeting new challenges (Gensitskiy, 2016). At present, with the popularization of the Internet and mobile Internet, hundreds of millions of massive, coarse-grained, and heterogeneous data generated every day largely contain information value that has not been valued or recognized so far. By mining the information value in these unstructured data and integrating market management strategies, we will be able to obtain new core and competitive value, establish competitive barriers, and then seize the opportunity in cross-border integration, rapid iteration, and decisive decision-making (Shoba et al., 2011). With the emergence of the concept of big data and the rapid development of the e-commerce market, some scholars have begun to pay attention to the integration of big data analysis and network marketing and the influence of their concept of enterprise marketing. Many scholars believe that sending is a revolution. Due to the huge data resources, various fields have started big data strategic plans. Leaders in various industries will quickly start such strategic plans. Think tanks of various governments have also sensed the commercial value contained in big data and have proposed government big data strategic plans (Yu and Zhou, 2021). On the one hand, network marketing will obtain a more professional basis for formulating strategies, and on the other hand, the potential value contained in big data will be the most realistic embodiment of marketization. In the book “Database Management Systems,” Professor Johannes Gehrke believes that big data enables enterprises to mine deeper data, obtain richer insights, and conduct a detailed analysis of network behavior through big data after each report overview (Gehrke, 2002). By integrating a marketing management strategy with big data, marketing organizations can make a substantial impact in key areas: combined with big data, we can determine that optimal online marketing spend across multiple channels and continuously optimize online marketing through testing, measurement, and analysis (Maksymiw, 2013).

The American Marketing Association defines consumer behavior as the dynamic interactive process of perception, cognition, behavior, and environmental factors, which is the behavioral basis for human beings to perform transaction functions in life (Bowers, 2017). Consumer behavior theory, also known as utility theory, studies how consumers allocate their income between goods and services so that maximizes the degree of satisfaction. To investigate consumer behavior, two analytical tools can be used: one is marginal utility analysis based on cardinal utility theory and the other is indifference curve analysis based on ordinal utility theory. The consumption process is an important part of consumer behavior, and it is worth noting that consumer behavior also includes post-consumption steps. The consumer purchase decision-making process includes causing consumption needs. The needs of consumers will lead to their interests or motivations, so as to search for relevant information, continuously improve their cognition of products or commodities, and collect relevant intelligence information around this thing, so as to establish consumer trust and make a comparative estimate. Combined with the pre-consumption preparations made above, based on certain knowledge judgments, after completing the purchase plan selection, it is finally converted into a decision to purchase goods. Consumers’ purchase feelings will affect their trust in the company or products and will form word-of-mouth communication. A good evaluation will play a positive role in the company’s future sales.

The factors that affected consumer behavior included cultural factors, which include not only the core of social members and culture but also subcultures. Cultural background can have an impact on an individual’s values. Different countries and economic levels result in different material and cultural levels and aesthetic standards, etc., which will affect consumers’ consumption management; social factors, contains family that includes the influence of family on individual purchasing behavior and the purchasing role of couples, also includes reference group and social class. A person’s purchasing behavior is also affected by groups, such as his friends or colleagues, and his social class is also one of the most important factors in purchasing decisions; personal factors, consumers’ economic status will strongly affect their consumption level. Different economic conditions will lead to different consumptions. Lower economic income can only carry out basic living consumption, while higher personal income can support high-end consumption improvements. The occupation and status of consumers are also important factors. Different occupations have different consumption preferences. Cultural categories, such as teachers, may be more enthusiastic about book consumption. In addition, consumers’ age, gender, personal personality, etc., are all personal factors that will affect consumer behavior and psychological factors include motivation, perception, learning, attitude, etc. Individuals first generate motivation and needs for consumption and then generate perception. The perception here is that after people filter out irrelevant information in life, some information produced a significant and rational influence, forming a perception.

Collecting relevant data on consumers’ actual purchasing behavior is an important means for business operators to evaluate the effectiveness of their integrated marketing strategies. To ensure that these actual purchase behavior data are objective and true, an important part is to measure consumer behavior (Kostelijk and de Mesquita, 2021). The measurement methods of traditional consumer behavior theory include indirect measurement, direct measurement, auxiliary measurement of the distribution system, etc.

Tourists constitute the main body of tourism activities. It is found that the research on tourist behavior mainly focuses on the research on the consumption level in tourism and the effect of various factors (Liu and Shi, 2021). Among them, in the aspect of tourist behavior research, foreign countries started related research earlier, and related research work has been carried out more than half a century ago, combining various disciplines to study tourist behavior in various dimensions and directions, and among them, the focus is on the behavior of tourism consumers (Tang et al., 2021). From an economic perspective, the study of tourism economic behavior through data from various dimensions began as early as the nineteenth century. The “Movement of Foreigners in Italy and the Money Consumed” published by Bodio of the Italian Political Statistics Bureau in 1899 used statistical methods to study the behavioral characteristics of tourists, and the related regularity of tourists’ travel behaviors is discussed. It is generally believed that “the movement of foreigners in Italy and the money they spend” is the original research on the economic aspects of tourists’ travel behavior. Since then, most tourism researchers at home and abroad have followed the tourism research path developed by Bodio to study tourism phenomena and have achieved rich research results. At the beginning of this century, some scholars began to predict and analyze tourism demand in combination with tourism demand models. They used econometric models, time series, logical inference methods, regression model methods, Delphi methods, and other methods to use tourism product prices (voices, tour guide expenses, transportation expenses, etc.), tourists’ personal income (or family income), tourist destination GDP, etc., are used as economic variables, and the influence of various factors and variables on tourists’ travel consumption behavior decision-making is analyzed. In these studies, the key representatives include Sila Kenyan and other scholars who believe that tourists’ decision-making includes three priorities, i.e., time, cost estimation, and attraction. Together they proposed a multi-variable destiny decision model. In addition, the important contribution of Wandorf and some scholars is to apply some methods in econometrics to tourism demand forecasting research.

At present, some domestic scenic spots consciously do some data analysis work, but usually only some small-scale questionnaire data, and there is no comprehensive and systematic collection of big data related to scenic spots through online platforms or other channels, and some scenic spots enterprises are excessively pursuing data. Accuracy, the amount of data are too small to be used. More scenic spots do not have the awareness of big data collection and analysis, let alone what big data will have a positive impact on the marketing of scenic spots. Therefore, this paper mainly studies and solves the above problems, establishes an analysis model for the number of tourists in scenic spots based on the relevant econometric theory and an analytical model for the impact of tourist types and secondary consumption in scenic spots. We put forward scientific and efficient scenic marketing management suggestions.

The rest of this article is arranged as follows: the second part describes the work related to tourist data collection; the third part proposes analysis methods and experimental results; the fourth part discusses the results; the fifth and final part. We put forward research conclusions, limitations, and possible strategies for tourism marketing. The innovation of this paper is that (1) when the model considers the error of explanatory variables and the possible omission of important variables, the parameters can still pass the significance test, that is, the model is stable, and all our suggestions and inferences are credible; (2) this paper is aimed to study the impact of various factors on the number of tourists in the Nanning Daming Mountain Scenic Area and the sales of secondary consumption items in the scenic spot through big data, improve the traditional econometric model, and adjust and optimize the linear correlation of some variables. The method of gradually adding variables and combining consumer theory to determine explanatory variables and build a more stable econometric model.

## Relate Work

### Data Introduction

This paper selects Nanning Daming Mountain Scenic Area as an example of big data analysis to illustrate the guiding role of big data information mining on the analysis of consumer behavior in scenic spots and the precise marketing of scenic spots. Tourist behavior of scenic spots mainly includes the choice of scenic spots (purchase Scenic spot entrance ticket) and secondary consumption in the scenic spot. The official website of Daming Mountain is the largest official e-commerce sales platform in the Daming Mountain Scenic Area. Therefore, in this article, we will study the sales of tickets on the official website and the consumption of secondary consumption items in the scenic spot on the official website. The sale items on the official website contain tickets for Daming Mountain Scenic Area, electronic coupons for secondary consumption items in the scenic area, such as Drifting Valley (additional payment is required to play) and restaurant food packages. The data used in this article are the actual sales data from the official website, provided by Guangxi Via Blue Information Technology Co., Ltd., and Nanning Daming Mountain Scenic Area. The data include the number of daily visitors to the website, online sales of scenic spots, online sales of consumer data, such as basic time and personal information of secondary consumption items (such as food and drifting valleys) in the scenic area, and web crawler technology to capture basic historical weather data in Nanning, Baidu index, etc., and this article-related data.

An important source of big data is the development of network and information technology, which provides enterprises with a large amount of full sample data. At the same time, online sales are the most important channel to record tourists’ consumption behavior data, and online sales are the sales ticket of Nanning Daming Mountain Scenic Area. The tourists who buy tickets online are individual customers, mainly young people, and group customers, such as agents. These online customers have become an indispensable source of tourists in the Daming Mountain Scenic Area in Nanning. The target of online sales obviously shows that Nanning Daming Mountain Scenic Spot has grasped the information needs of modern people, and analyzing online tourists is bound to provide valuable advice for the future development of Nanning Daming Mountain Scenic Spot. The advantages of sample data are that it is true, accurate, and time-sensitive. The data in this research come from the online sales system of the Daming Mountain Scenic Area in Nanning. The system stores the complete data on the consumption behavior of tourists on the official online platform of Daming Mountain. These data will not be distorted by respondents because of the data. Online payment has become the mainstream of consumption, and the trend of sample data objects obtained through it is controllable. At present, most people have used the Internet or even mobile phones to make payments, and the data trend is relatively stable. Although the data in the sample time have a time trend, the trend is controllable, and the conclusion is that there is no model error problem of time extrapolation.

### Model Construction

We established an analysis model based on the personal basic information of online ticket-buying tourists, historical weather, search engine influence, and other big data and used these big data to study the scenic tourism consumer behavior characteristics of online ticket-buying tourists in Nanning Daming Mountain Scenic Area.

We hypothesized that tourist travel plans are affected by the basic weather conditions; consumers’ consumption behavior is greatly influenced by the guidance of the search engine and the quality of the official website of the scenic spot. There are differences in the consumption behavior and consumption ratio of different types of tourists in the scenic spot, and the consumption ratio of parent-child groups is higher.

This study believes that the influencing factors that affect which scenic spots potential tourists ultimately choose to travel include the quantity and quality of scenic website traffic, the influence strength of scenic search engines, weather quality, and others. We chose to send some variables only because they have a theory behind them and the data are easier to obtain.

Initial Model Construction of Tourist Quantity in Scenic Spots: To better expand the source of tourists in Nanning Daming Mountain Scenic Area, we need to study the main factors based on the customer data generated in the past that affect tourists to visit Nanning Daming Mountain Scenic Area—basic weather conditions, weather quality, the number of website visitors, the influence of scenic spots on search engines, etc. In this article, we will analyze various main factors that will affect the number of tourists in the Daming Mountain Scenic Area and make estimates and corresponding judgments on them. According to the model’s hypothesis, an initial equation can be built as Eq. (1).


(1) $$Y=\omega_{0}+\omega_{1}\,I\,P+\omega_{2}\,P\,V+\omega_{3}\,U\,V+\omega_{4}\,B\,D+\omega_{5}\,P\,C+\omega_{6}\,M\,o+\omega_{7}\,W\,K+\omega_{8}\,B\,o+\omega_{9}\,l\,a+\omega_{10}\,A\,Q\,I+\omega_{11}\,P\,M\,2.5+\omega_{12}\,P\,M\,10+\varepsilon$$

Where Y is the number of tourists; IP is the daily internet protocol (IP) number of visiting the official website of Nanning Daming Mountain Scenic; PV is website visiting number; UV is read frequency of website; BD is daily Baidu index; PC is PC part of Baidu index; Mo is the mobile part of Baidu index; WK is a dummy variable for weekends, where a value of 1 indicates a weekend, and a value of 0 indicates a weekday; Bo is website bounce rate, an important indicator of website traffic quality; la is the average visit time of the website; AQI is air quality index; PM2.5 is air concentration of particulate matter with aerodynamic equivalent diameter less than or equal to 2.5 microns; PM10 is air concentration of particles with aerodynamic equivalent diameter less than or equal to 10 microns; ε is the error term of a normal distribution with zero mean and homoscedasticity that include other random factors that may affect the number of customers; and ω is parameters to be estimated. Due to the highly linear correlation of some variables, there will be biased estimates in the process of model estimation, and the significance test of variables and the predictive function of the model are meaningless. Therefore, this model is not the final model and needs to be revised continuously. Before the model is revised, we need to perform a multicollinearity test of the model. The diagnostic methods used for multicollinearity problems include auxiliary regression analysis, variance inflation factor (VIF) analysis, and so on.

## Methods and Results

### Research Methods

Auxiliary regression, also known as dependent regression, is to use a certain explanatory variable as the explained variable and the other explanatory variables as the explanatory variables to perform regression. According to the goodness of fit of the auxiliary regression model and the *F*-test statistic, the degree of multicollinearity can be judged. There are generally two methods for diagnosing multicollinearity. The first diagnostic method is that if the *F*-test of the auxiliary regression is significant, it can be considered that there is a significant multicollinearity problem. The second method is that for partial k, there is R2<Rk2, we can judge that multicollinearity problem. For all k, there are R2<Rk2, we can judge that there is a significant multicollinearity problem. For the original model, *R*^2^=0.7253,*F*=23.23, the regression model is significant, each explanatory variable is used as the explained variable in turn, and the remaining explanatory variables are used as the explanatory variables for regression, and the goodness of fit and *F*-test statistic of the auxiliary regression is observed (for results see Table 1).

**TABLE 1:** Fitting goodness and F test statistic of auxiliary regression.

Explained variable	AQI	PM2.5	PM10	PV	UV	IP	Bo	la	BD	PC	Mo
R^2^	0.9800	0.8762	0.9709	0.9812	0.9985	0.9956	0.2903	0.3562	1.000	1.000	1.000
F	478.99	80.30	450.32	280.35	5322.23	5369.12	6.91	5.23	–	–	–

We found that when Baidu, PC, Mobile, etc., are used as explained variables, the goodness of fit is close to 1, and the F statistic is infinite. It can be seen that there may be a high degree of multicollinearity between Baidu, PC, Mobile, and other explanatory variables.

According to the principle of econometrics, when the explanatory variables exhibit multicollinearity, the variance of the parameter estimator will become larger, so the VIF can be defined as:


(2) $$V\,I\,F\,\left(b_{k}\right)=\frac{V\,a\,r\,\left(b_{k}|X\right)}{V\,a\,r_{1}\,\left(b_{k}|X\right)}$$

Where *Var*(*b*_*k*_|*X*) is the conditional variance of parameter estimator *b_k*for Y to X in multiple regression, *Var*_1_(*b*_*k*_|*X*) is the conditional variance of parameter estimator *b*_*k*_for Y to X in single regression. The VIF is a measure of the degree to which the variance of the parameter estimator is enlarged after adding explanatory variables on the basis of univariate regression.

It can be shown that the VIF conforms to the following relationship:


V⁢I⁢F⁢(bk)≈11-Rk2

Where Rk2 is the goodness of fit of the regression of the explanatory variable X to the remaining explanatory variables.

If X has nothing to do with the rest of the explanatory variables, Rk2=0, *VIF*(*b*_*k*_)=1; If the relationship is collinear, Rk2=1, *VIF*(*b*_*k*_)= + .

The results (not shown) from our calculation show that the significant variables include the number of IPs visiting the official website of Nanning Daming Mountain Scenic Area every day, the number of website page views, the number of website visitors, Baidu index, and Baidu index mobile terminal, etc. At the same time, we conducted a VIF test on all variables introduced in the initial model (see Table 2).

**TABLE 2:** VIF test results.

Variable	VIF	1/VIF
UV	550.98	0.00181
IP	545.45	0.00183
AQI	49.32	0.2028
PM10	40.12	0.0249
BD	42.32	0.0236
Mo	33.66	0.02971
PV	31.09	0.03216
PM2.5	9.05	0.11050
Bo	1.60	0.63043
la	1.51	0.66122
Mean VIF	131.50	

As shown in Table 2, the one with the smallest VIF value of all variables introduced in the final model appears on the average visit duration, which is 1.51. The tolerance value method is also one of the methods for diagnosing multicollinearity. The tolerance value Tol is the reciprocal of the VIF value (i.e., Tol = l/VF). The tolerance value is between 0 and 1, and the largest value is 0.66122. According to the tolerance value method, combined with the tabular data, there is no multicollinearity in the three variables of average access duration, bounce rate, and PM2.5 index.

The multicollinearity of the model data was confirmed using auxiliary regression analysis and VIF analysis. When multicollinearity is serious, the parameter estimator is unreliable, and the parameter conditional variance estimation value becomes large, resulting in an unreliable single-parameter *t*-test. Therefore, the multicollinearity problem needs to be corrected. This paper adopts the stepwise regression method to verify it.

There are several consequences of multicollinearity. The parameter estimator does not exist under complete collinearity, but in most cases complete collinearity is rare, and basically, a certain degree of collinearity is more common. Under multicollinearity, the economic meaning of the parameter estimator is unreasonable, and the significance test of the variable will be difficult to pass, which will eventually lead to the loss of the predictive function of the model. One variable is selected for regression calculation in each group variable. Considering that air quality may have an impact on tourists’ travel, dummy variables are selected for air quality to replace, specifically the dummy variable (Pollution) for weather quality with mild pollution and above. A value of 0 indicates that the weather quality of the day is excellent or good. Among the multicollinearity correction methods, the step-by-step regression method usually includes the step-by-step addition method and the step-by-step deletion method. This article tries both methods first. In both methods, we set the probability threshold for the variable to pass the *t*-test to be 0.05. In step-by-step delete method, we delete variables that include Bo (*p* = 0.6978), PM10 (*p* = 0.4455), PM2.5 (*p* = 0.1519), AQI (*p* = 0.4082), and la (*p* = 0.2658), due to not stratify threshold. In step-by-step regression, due to satisfy threshold, we add variables that include PV (*p* = 0.0000), PC (*p* = 0.0012), and BD (*p* = 0.0000). Through regression calculation, we can obtain the final regression format.


Step-by-step⁢delete⁢method:peolpe=57.51-1.671⁢PC+2.373⁢IP+0.4705⁢BD+0.9960⁢PV-2.4⁢UV;

Step-by-step⁢add⁢method:people=67.92+0.08739⁢PV-1.775⁢PC+0.4353⁢BD.

From the stepwise regression method, it can be seen that there are more variables that are gradually deleted and retained and fewer variables that are gradually added. In the gradual deletion method, the remaining variables are basically the variables of network traffic quality and Baidu index. In the gradual addition method, the remaining variables are also the variables of network traffic quality and Baidu index. However, observing the goodness of fit of these two methods, the stepwise deletion method is higher, so it is possible that the stepwise deletion method can better describe the entire system. Considering the significance of economic variables, this paper still properly retains the typical variables in various types of variable families according to the economic principle after adopting the step-by-step deletion method and adding dummy variables for weekends and weather quality at the same time. The revised model is:


(3) $$\mathrm{people}=\omega_{0}+\omega_{1}\,\mathrm{IP}+\omega_{2}\,\mathrm{BD}+\omega_{3}\,\mathrm{WK}+\omega_{4}\,\mathrm{Bo}+\omega_{5}\,\mathrm{Pollution}+\varepsilon$$

However, this paper pays more attention to the linearity and unbiasedness of the parameter estimators, and a small number of non-validities do not affect the main conclusions. Most of the variables finally passed the significance test, and the instant model has a small number of multicollinearity problems, and the conclusions on the parameter estimators are not affected.

### Data Analysis

Preliminary sorting out of the big data related to scenic spots (extraction of valid data and removal of noise) is the first step in data analysis. The data collected at hand cannot be directly used for analysis due to the inconsistent time span of the data, the inconsistent data storage structure before and after the year, and the inconsistent range of information records before and after the year. To this end, it is necessary for us to filter the original data and retain the data that is highly correlated with the number of customers and retains relatively complete information for analysis. Considering that some of the original data are missing on some days, and the overall data are very helpful for analyzing consumer behavior, the missing data are linearly filled to obtain relatively complete data.

### Big Data Analysis of Historical Weather in Scenic Spots

When tourists decide on the destination of tourist attractions, they will also be affected by the local weather and air quality of the scenic spots, which is a very important influencing factor in the process of tourists’ tourism consumer behavior. Climate and weather-related variables, such as temperature, precipitation, wind speed, are important factors that affect tourists’ destination choices. From the statistical analysis of big data, it can be seen that the frequency of rainy days and cloudy days in the basic weather indicators of Nanning is relatively high, indicating that the historical weather in Nanning is relatively good and there are more rainy and cloudy days (not shown in here). From the average humidity in Nanning, it can be seen that the annual average temperature is maintained at 14°C, the climate is relatively mild, the summer is long, and the winter is short. Judging from the highest temperature and lowest temperature in Nanning every month, Nanning’s monthly temperature is generally not low, which belongs to a typical subtropical climate.

### Big Data Analysis of Tourist Ticket Purchases (Such as Historical Repeat Purchase Analysis)

**FIGURE 1:**
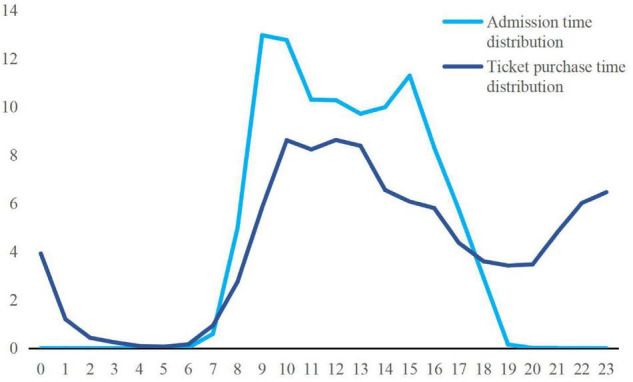
Single-day time distribution of ticket purchase and admission in Nanning Daming Mountain Scenic Area.

The original data collected in this article contain the data of each oral ticket transaction process of Nanning Daming Mountain Scenic Area, such as ticket purchase time, entry time, number of tickets purchased, type of oral ticket, and personal information (name and mobile phone). In this paper, the method of frequency analysis is used to preliminarily organize the collected big data and analyze the behavior of tourists buying tickets. Nanning Daming Mountain Scenic Area can launch time-sensitive marketing activities, such as short-term spike promotions for tourists who buy tickets instantly at different peak periods during the peak period of a single day to carry out bundled publicity and launch a seckill purchase on the hour, it will be possible to maximize the benefits (Figure 1). Nanning Daming Mountain Scenic Area can strengthen the official website ticket bundled restaurants or drifting valley limited-time preferential packages before the peak season in August and every weekend, and increase the total amount of ordering online in advance by single tourist. In the off-season, limited-time sale discounts can be used for tickets and other methods are used to recruit clever customers, so that the number of tourists in the scenic spot in the off-season does not decline too much and the annual income can be maximized. Tickets for the Daming Mountain Scenic Area in Nanning are mainly short-term ticket sales. This is a good revelation for the short-term peak expectations of the Nanning Daming Mountain Scenic Area: for the upcoming peak period, the Nanning Daming Mountain Scenic Area should strengthen the early warning and do a good job of warning of various accidents during the peak period. The behavioral characteristics of today’s consumers when conducting online consumption are as follows: first, one person is responsible for bribing tickets, such as in the case of couples or families. Second, because of the popularity of mobile payment, tourists who travel together to buy their own tickets directly on their mobile phones, so there are more cases where a single person buys a ticket. The sale items on the official website of Nanning Daming Mountain Scenic Area include electronic tickets and electronic coupons for restaurant meal packages. Specifically, tourists can purchase the Daming Mountain Scenic Area combined ticket (such as Daming Mountain Scenic Area and Drift Valley) on the official website or just buy the ordinary ticket of the Daming Mountain Scenic Area. At the same time, according to the type of tourists, tickets are divided into parent-child joint tickets and individual tickets, and individual tickets are divided into two types, i.e., student tickets and adult tickets, and there are some free tickets.

### Big Data Analysis of Tourist Visits to the Official Website of the Scenic Spot

Website technicians record the browsing behavior of each visitor to the official website of the scenic spot by writing professional access statistics programs or by using cloud statistical technology services, such as Baidu statistics, and form a complete full sample access data for the scenic spot. Operators continue to optimize website content settings, functional design, and development to provide a lot of scientific support. The professional network technology department of the scenic spot will conduct targeted analysis and statistics based on these refined access data, dig out the subtle behavioral characteristics and laws of customers visiting each column, explore the behavioral activities of target customers in the scenic spot, and refine them, and the tourism needs of potential target tourist out of scenic spot potential target tourists out of the scenic spot. Combine these analysis results with the business strategy of the scenic spot and further revise the scenic spot’s operating strategy and promotion plan. Generally speaking, indicators to measure the number of visits to a website include PV, UV, IP numbers, etc., and indicators that reflect the quality of a website visit include bounce rate, visit time, and so on. The bounce rate generally counts the behavior of visitors closing the website after only visiting one page, which reflects the traffic quality of the website. For a website, the lower the bounce rate, the better, indicating that the column settings and functional modules of the website meet the needs of tourists so that they can browse the website for a long time.

We selected the data of 2013 and 2014 for statistical analysis (not shown in this article), and the results show that the daily visitor volume of the scenic site is consistent with the trend of the mad season of the scenic spot. In 2014, the number of daily PV on the website during the peak period was less than that in 2013, which may reduce the number of tourists in the Daming Mountain Scenic Area in Nanning, which is worth our warning. It can be seen from the bounce rate of the scenic official website that reflects the quality of the website, the bounce rate is lower in summer and higher in winter, indicating that people are more willing to pay attention to the official website in summer. However, due to the altitude, Daming Mountain in winter often snows. It is a peculiar scenery in Nanning, where the weather is hot. The official website of Daming Mountain has a low number of visitors and fewer tourists in winter, indicating that the scenic spot does not focus on excavating Daming. Due to the advantages of tourism resources in the mountain scenic area in winter, tourists do not have the consumption needs to go to the Daming Mountain scenic area to watch the snow in winter.

### Big Data Analysis of Search Engine Massive Search Behavior (Baidu Index)

The Baidu index reflects the macro statistics of the massive search behavior of scenic spot-related keywords in the Baidu search engine. It can reflect the size of the search engine for scenic spot-related keywords, the ups and downs over a period, and the changes in related news and public opinion. The scenic spot optimizes the new media promotion and official website marketing plan. According to the keyword “Daming Mountain Scenic Area in Nanning” as the search target, we obtained the Baidu index and plotted the overall trend, PC trend, and mobile trend in chronological order.

Baidu index of “Nanning Daming Mountain Scenic Area” has a certain periodicity. The period is 1 year. The high peak period is concentrated in July and August each year, and the low point period is concentrated in January each year. At the same time, the Baidu index shows a slight growth every year. It can be seen from this that the Baidu index is basically the same as the peak period of tourists entering the park. Summer is the start season for tourists, and winter is the low season for tourists.

In 2014, the number of search studies for “Nanning Daming Mountain Scenic Area” on the mobile client began to be higher than that on the PC, indicating that the mobile Internet is gradually becoming the mainstream, which will also attract tourists in the future. As the main channel, Nanning Daming Mountain Scenic Spot can try to launch the Nanning Daming Mountain Scenic Spot app, WeChat website, etc., to guide tourists to use the mobile payment for quick consumption.

### PM2.5 Air Quality Data Over the Whole Year

Many consumers also take the local climate and air quality as an important consideration when choosing a scenic spot for tourism. Tourism is an activity that is both relaxing and healthy, and if there is a serious fog in the scenic spot. If the weather is bad, tourists will have greater pressure and resistance, which will have a negative impact on the decision to go to the scenic spot for consumption. This part analyzes the historical weather quality of Nanning based on the big data of historical air quality in Nanning.

**FIGURE 2:**
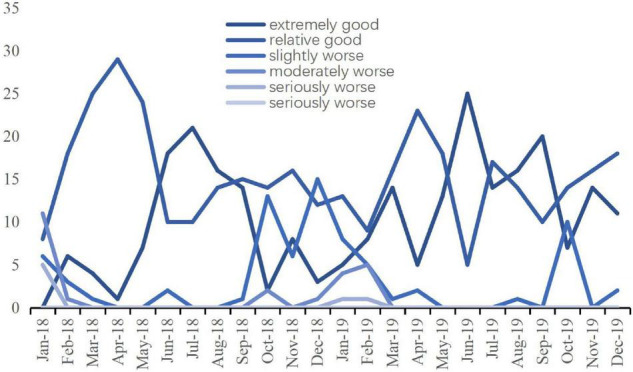
Historical air quality distribution in Nanning province.

This paper collects the big data of historical air quality in Nanning from 2018 to 2019 through crawler information technology and conducts frequency statistical analysis. The statistical analysis results are shown in Figure 2. We found that the historical air quality in Nanning was generally good, with the distribution of good and good in the majority. The air quality in Nanning has a certain seasonality. The air quality in summer is relatively good, and the air quality is mostly excellent. The air quality in winter is slightly worse, and the days with air pollution levels are slightly more. This may be related to the lush vegetation and strong air purification ability in Nanning in summer.

### Analysis of Secondary Consumption Big Data in Scenic Spots

In addition to choosing the destination of the scenic spot and purchasing the scenic spot ticket, the consumer behavior of tourists in the scenic spot also includes the consumption behavior in the scenic spot. Therefore, we extracted the sales data of the official website in 2019 for the rafting Taniguchi tickets and the e-coupons of restaurant food packages in the scenic area for analysis and research.

Meanwhile, according to the original sales data on the official website in 2019, a total of 9,067 student tickets were sold, 2,002 single adult tickets, 453 parent-child child tickets, and 413 parent-child adult tickets. Among them, the parent-child children in Table 3 represent the number of children who purchased the Tourism project of Drift Valley tickets among the children who purchased parent-child tickets; the parent-child adults represented the number of adults who purchased the parent-child tickets who purchased the Drift Valley. In Table 4, parent-child children represent the number of children who purchased parent-child tickets and the number of children who purchased scenic restaurants; and parent-child adults represented the number of adults who purchased parent-child tickets, who purchased food packages in scenic restaurants. The relationship between tourist types and secondary consumption in scenic spots is shown in Table 3.

**TABLE 3:** Rafting class distribution within the scenic valley visitors.

	Student	Single adult	Parent-child	Parent-adult
2019/1	276	19	9	10
2019/2	207	32	7	6
2019/3	280	86	6	10
2019/4	384	47	9	13
2019/5	607	73	11	15
2019/6	1,574	136	18	23
2019/7	1,177	237	33	39
2019/8	865	198	21	22
2019/9	384	23	9	12
2019/10	232	39	6	10
2019/11	107	11	5	97
2019/12	165	5	7	9
Total	6,258	906	141	176

**TABLE 4:** The distribution of secondary consumption visitors in the dining area.

	Student	Single adult	Parent-child	Parent-adult
2019/1	68	36	22	16
2019/2	49	25	15	13
2019/3	78	44	12	10
2019/4	102	34	17	12
2019/5	98	45	15	11
2019/6	224	129	23	15
2019/7	256	193	58	59
2019/8	236	172	56	70
2019/9	49	84	22	13
2019/10	35	54	23	11
2019/11	43	67	15	12
2019/12	25	33	23	18
Total	1,263	916	301	260

## Results and Discussion

### Stability Check

To verify the robustness of the results, this paper changed some variables to other substitute variables in turn and then re-regressed the regression analysis to observe the changes in the coefficients and statistics.

To verify whether the influence of the number of tourists visiting the official website of the scenic spot on the number of tourists is stable, the author changed the IP number (IP) of visiting the official website to the number of PV and the UV, respectively, and carried out regressions, respectively. The results are shown in Table 5. Change the Baidu index (BD) to the PC terminal (PC) and the mobile terminal (Mobile), respectively, and perform regression, respectively. Replace the dummy variable (pollution) of air quality with the AQI index and PM2.5 and perform regression, respectively. Considering that web browsing and search may have a greater relationship with future tours, the IP number and Baidu index are replaced by their lag term L. IP and L. Baidu and then return.

**TABLE 5:** Stability testing.

	People	People	People	People	People	People	People
IP			0.452***	0.275***	0.269***	0.275***	
			(7.07)	(5.61)	(4.87)	(4.94)	
L.IP							0.391***
							(8.01)
PV	0.0582***						
	(5.76)						
UV		0.282***					
		(5.02)					
Baidu	0.104***	0.144***			0.158***	0.154***	
	(1.91)	(2.66)			(2.99)	(2.86)	
L.Baidu							0.0742
							(1.61)
PC			−0.327				
			(−1.50)				
Mobile				0.234***			
				(3.89)			
Weekend	70.07***	71.15***	75.84***	63.42***	69.35***	69.50***	67.78***
	(6.28)	(6.14)	(6.55)	(5.55)	(5.95)	(6.00)	(6.68)
Bounce	−137.7	−308.1**	−331.1**	−304.7**	−289.6*	−295.8*	−365.9***
	(−0.90)	(−2.03)	(−2.12)	(−2.08)	(−1.92)	(−1.95)	(−2.69)
pollution	36.83	31.26	8.411	32.39			40.97
	(1.05)	(0.86)	(0.22)	(0.93)			(1.26)
AQI					0.502		
					(1.53)		
PM2.5						0.401	
						(0.91)	
Constant	1.966	65.75	147.6*	68.09	31.54	49.26	89.50
	(0.02)	(0.82)	(1.72)	(0.89)	(0.38)	(0.60)	(1.27)
F	34.96	31.56	29.15	35.61	32.87	32.05	43.18
R^2^	0.653	0.629	0.610	0.657	0.639	0.633	0.701
AIC	10565	1063.0	1067.7	1055.3	1060.5	1062.1	1029.3
BIC	1072.1	1078.6	1083.5	1070.9	1076.0	1077.6	1044.8
Root MSE	48.799	50.428	51.685	48.507	49.78	50.18	44841

T statistics in parentheses *p < 0.1, **p < 0.05, ***p < 0.01.

From the regression results, the number of visitors to the website has a positive impact on the number of visitors and passes the significance test, the only difference is the specific numerical value. It shows that the increase in the number of visitors to the website can significantly increase the number of real tourists in the Daming Mountain Scenic Area in Nanning. The coefficient of the Baidu index is negative but not significant after changing to the PC terminal, and the coefficient is positive and significant after changing to the mobile terminal, which once again shows that the mobile terminal will replace the PC terminal and become the mainstream of Baidu search in the future. In the future, Nanning Daming Mountain Scenic Area should gradually pay attention to the potential customers of the mobile terminal, increase the investment in mobile terminal technology and advertising, and increase the market share of the mobile terminal. Air quality is still insignificant after changing other variables, indicating that air quality in Nanning may not have become a factor restricting tourists’ travel, but future warnings must also be strengthened to take early warning measures for extreme weather. The coefficient of the lag term passes the test, indicating that increasing the number of tourists will indeed increase the number of tourists by increasing the number of tourists searching or browsing the website. The overall model passes the stability test, which proves that our conclusions are robust.

### The Increase in the Number of Tourists on the Official Website of the Scenic Spot Can Increase the Number of Tourists in the Daming Mountain Scenic Area in Nanning

The scenic spot should continue to expand the influence of the official website, add keywords, and add information about customer relations, so that the scenic spot becomes a beautiful business card for local tourism. Scenic spots should carry out effective cooperation and support in product development, marketing, and market strategy. Scenic spots and scenic spots can strengthen various forms of cooperation with travel agencies and tourism websites with large business volumes in the city, establish strategic cooperation alliances, and provide enterprises in the alliance with better services and prices, so as to achieve the purpose of winning.

The increase in the massive search behavior of search engines can significantly increase the number of tourists in the Daming Mountain Scenic Area in Nanning. The macro indicators of the massive search behavior of search engines directly affect the actual number of tourists in scenic spots. With the popularization of the Internet and the development of the mobile Internet, the official website of the scenic spot and the related information of the scenic spot on the search engine have played a role in stimulating the tourist motivation of tourists. It reflects the tourists’ travel expectations and motivations. When there are a large number of search behaviors for related keywords of a certain scenic spot, it reflects that the number of potential tourists in the scenic spot or the scenic spot is relatively large, which is an important indicator of the popularity of a scenic spot among potential tourist groups.

### Tourists Prefer to Visit Nanning Daming Mountain Scenic Area on Weekends

The popular science feature of Nanning Daming Mountain Scenic Area determines that minors must be an indispensable source of tourists. In addition to the previous analysis, the student customer group is the main source of tourists in the Nanning Daming Mountain Scenic Area. There is a small peak of tourism in the Daming Mountain Scenic Area in Nanning. Since tourists prefer to visit Nanning Daming Mountain Scenic Area on weekends, Nanning Daming Mountain Scenic Area should focus on launching new varieties and new projects on weekends to better attract more tourists, especially for specific customer groups.

Adding content that customers are interested in on the official website can greatly increase the number of tourists in the Daming Mountain Scenic Area in Nanning. Due to the high bounce rate of the official website of the scenic spot, it is necessary to consider whether to update the keywords when designing the website, whether the creative writing is excellent, consider the access speed of the website, and study whether the user can find the required content after entering the website (visit URL settings) and whether the content of the website is attractive to users in line with the user experience. The emphasis on website design is the emphasis on the visitor experience. In particular, the official website of the scenic spot should design the column content of the website according to the needs of different tourist groups. At the same time, the website should also have tourist attractions information, traffic information, entertainment information, travel common-sense information tips, travel healthcare information, and other travel-related weather and road information.

At present, the weather quality in Nanning has little impact on tourists. Since the weather quality in Nanning has been good in recent years, the weather with severe pollution is relatively rare, and the weather quality has not yet become a strong constraint restricting the travel of Nanning citizens. Therefore, the analysis results show that the impact of weather quality on the number of tourists is not significant. But this does not mean to relax the vigilance of polluted weather. Since Wuba is a hot topic in the country’s current society, many domestic scholars believe that Wuling has become an important factor that cannot be ignored in the process of potential tourists’ destination selection. In fact, the bad foggy weather will inevitably lead to the loss of potential tourists, which may have a negative effect on the income of scenic spots.

We should increase the parent-child section and improve the quality of website design details. According to the previous analysis, it can be seen from the bounce rate of the official website of the scenic spot that reflects the quality of the website, the bounce rate is lower in summer, and the bounce rate is higher in winter. For every 1% increase in the bounce rate of the website, the number of tourists will decrease by about 3, indicating the number of IPs. For every 100 bureaus, the number of tourists to Daming Mountain in Nanning can increase by 28.1 people. For every 100 increase in Baidu index, the number of tourists in the Daming Mountain Scenic Area in Nanning can increase by 14.9 people.

Therefore, for the secondary consumption projects in the scenic area, combined with big data analysis, we should strengthen the design details of the official website, reduce the bounce rate, and at the same time increase the exposure of the Daming Mountain Scenic Area in the network atmosphere, thereby increasing the Baidu index and website traffic. Moreover, setup a special column for the parent-child tourist group or revise the website to highlight the parent-child interest column in the scenic spot, because the parent-child tourist group has a higher proportion of food consumption in the scenic spot, if the parent-child group’s website access big data can be improved, it will directly affect the number of parent-child tourists in the scenic spot, which will greatly increase the turnover of restaurants in the scenic spot.

### Limitations

It should be noted that although the overall coefficient of determination of the model is not high, it does not mean that the explanatory power of the model is not strong. Because the official website ticket sales data and PV data have inconsistent time spans due to the need for commercial confidentiality. Considering the completeness of the data, we can only select the time period with complete data for analysis, resulting in a serious reduction in the number of our samples.

## Conclusion

This paper takes the big data related to the scenic spot as the research object and explores the relationship between various subdivided big data and the number of tourists in the scenic spot. The difference and influence of the consumption behavior of the secondary consumption items in the scenic spot, to find the potential for the business growth of the scenic spot, so as to promote the continuous and stable growth of the scenic spot’s sales and tourism economy. This paper uses related theories and analysis methods, such as consumption behavior, big data, and tourism consumer behavior. The calculation of the contribution rate of type data to the number of tourists in the scenic spot and the difference analysis of the secondary consumption items of different types of tourists in the scenic spot are carried out. The main research results are:

1. A multi-objective analysis model is established based on the relevant theories of econometrics, and an optimization scheme is proposed after the multicollinearity diagnosis of the model. The model includes five elements, i.e., big data of tourists’ historical online ticket purchases, big data of tourist visits on the official website of the scenic spot, big data of massive search behaviors of search engines, and big data of local PM2. The selection of elements should not only conform to the empirical formula but also take into account the actual situation of the scenic spot, which can more realistically reflect the actual impact of the consumption behavior factors of big data on the sales of the scenic spot.
2. We have established a data envelopment analysis (DEA) model of the difference and influence of different types of tourists’ consumption behavior in scenic spots and studied the consumption behavior characteristics of different types of tourists when they purchase secondary consumption items in scenic spots.
3. The econometric model is used to analyze the big data, adjust the linear relationship of some variables, adopt the method of gradually adding variables combined with the consumer theory, and determine the number of daily tourists as the explained variable, the number of IP, Baidu index, and the virtual value of the weekend. Variables, bounce rate, and dummy variables for air pollution were used as explanatory variables. The increase in the number of tourists visiting the official website of the scenic spot can significantly increase the number of tourists in the Daming Mountain Scenic Area in Nanning; the increase in the massive search behavior of search engines can significantly increase the number of tourists in the Daming Mountain Scenic Area in Nanning; tourists prefer to visit the Daming Mountain Scenic Area in Nanning on weekends; adding content that customers are interested in on the official website can greatly increase the number of tourists in the Daming Mountain Scenic Area in Nanning; the pros and cons of the former Nanning weather quality have little impact on tourists; and the type of tourists has an impact on the secondary consumption in the scenic spot.

## Data Availability Statement

The raw data supporting the conclusions of this article will be made available by the authors, without undue reservation.

## Author Contributions

All authors listed have made a substantial, direct, and intellectual contribution to the work, and approved it for publication.

## Conflict of Interest

The authors declare that the research was conducted in the absence of any commercial or financial relationships that could be construed as a potential conflict of interest.

## Publisher’s Note

All claims expressed in this article are solely those of the authors and do not necessarily represent those of their affiliated organizations, or those of the publisher, the editors and the reviewers. Any product that may be evaluated in this article, or claim that may be made by its manufacturer, is not guaranteed or endorsed by the publisher.
